# Complement and Coagulation Cascades Pathway was Inactivated in HIV-Associated Colorectal Cancer: Results from a Proteomics Study

**DOI:** 10.7150/jca.124804

**Published:** 2026-01-14

**Authors:** Shixian Lian, Lei Li, Yuexiang Yang, Siyuan Liu, Shu Song, Lijun Zhang

**Affiliations:** 1 Shanghai Public Health Clinical Center, Fudan University, Shanghai 201508, China.; 2 Shanghai ninth People's Hospital, Shanghai Jiaotong University School of Medicine, Shanghai 200011, China.

**Keywords:** HIV, colorectal cancer, proteomics, ribosome, complement and coagulation cascades

## Abstract

**Background**: Colorectal cancer (CRC) remains a leading cause of global cancer-related morbidity and mortality. Human Immunodeficiency Virus (HIV)-1 infection worsens colorectal cancer (CRC) outcomes.

**Methods**: To investigate mechanisms, we conducted Tandem Mass Tag proteomics on tumor (C) and adjacent normal tissues (A) from five HIV-positive (HIV+) and four HIV-negative (HIV-) CRC patients. Four comparisons were analyzed: HIV+C vs HIV+A (differentially expressed proteins, (DEPs)-1), HIV-C vs HIV-A (DEPs-2), HIV+A vs HIV-A (DEPs-3), HIV+C vs HIV-C (DEPs-4) (|fold change| ≥ 2, p < 0.05). The DEPs specifically affected by HIV (DEPs-5) underwent KEGG pathway enrichment analysis. The relative abundance of pathway-associated DEPs was compared with the data from CPTAC database. Key DEPs were validated by western blot/immunohistochemistry.

**Results**: We identified 749 (DEPs-1), 431 (DEPs-2), 4 (DEPs-3), and 21 (DEPs-4) DEPs. After excluding DEPs common to other comparisons, 592 HIV-specific DEPs (410 up-, 182 downregulated) were identified. KEGG enrichment revealed top altered pathways: upregulated ribosome (40 proteins) and downregulated complement and coagulation cascades (CCC pathway; 24 proteins). Comparison with the CPTAC database showed that HIV infection significantly increased the expression of upregulated DEPs but only slightly decreased the expression of downregulated ones. Downregulation of key CCC pathway proteins (C8B and SERPINA1) was confirmed by western blot and immunohistochemistry, respectively.

**Conclusion**: HIV-associated CRC exhibits distinct proteomic alterations, particularly ribosome and CCC pathway dysregulation. *C8B* and *SERPINA1* are potential biomarkers for HIV-CRC.

## Introduction

Colorectal cancer (CRC) is a leading cause of cancer-related mortality in China, ranking second in incidence 1. Both its morbidity and mortality rates exhibit persistent upward trends nationally 2. Despite therapeutic advances, CRC prognosis remains suboptimal, with an approximate 5-year mortality rate of 12.8% 3. Metastatic disease portends a particularly poor outlook, demonstrating ≤ 50% for 5-year survival despite surgical intervention 4. Etiological factors include genetic predisposition, environmental exposures, and chronic inflammatory states 5. Pathogenic microorganisms, including viruses, further contribute to carcinogenesis through sustained inflammation and dysregulated cytokine signaling 6, 7. Previous studies have demonstrated a heightened CRC incidence among individuals with HIV-1 infection versus the general population 8-10. Clinically, HIV-positive CRC patients present with higher tumor grades, more aggressive disease progression, more advanced staging at diagnosis, and poorer survival compared to their HIV-negative counterparts 6, 8, 9.

The widespread adoption of highly active antiretroviral therapy (HAART) has significantly prolonged survival in people living with HIV (PLWH) over recent decades 10, 11. This extended lifespan correlates with rising colorectal cancer (CRC) incidence within this population. However, the pathophysiological mechanisms driving HIV-associated CRC carcinogenesis remain poorly understood. Consequently, elucidating the molecular interplay between HIV infection and CRC pathogenesis is critical, both to uncover underlying mechanisms and to address therapeutic disparities in HIV-infected cancer patients 12.

To address this gap in mechanistic understanding, we performed comparative proteomic profiling of tumor tissues and adjacent normal mucosa from CRC patients with or without HIV co-infection. Our analysis uncovered pronounced alterations in ribosomal proteins and complement/coagulation cascade components. This work establishes high-throughput proteomics as a powerful approach for delineating pathogenic drivers and identifying candidate diagnostic biomarkers in HIV-associated CRC.

## Materials and Methods

### Ethics approval

This study was approved by the Ethics Committee of Shanghai Public Health Clinical Center (Protocol 2019-S035-02), operating under the principles of the 2013 Helsinki Declaration. All participants provided written informed consent. Tumor tissues and matched adjacent normal colonic specimens were obtained from residual clinical specimens after pathological diagnosis. Relevant clinical parameters, including demographic and histopathological data, were retrospectively retrieved from institutional electronic medical records.

### Experimental design

Motivated by known influences of HIV-1 on carcinogenesis and treatment response, this study aimed to compare the proteomic profiles of colorectal malignancies from HIV-positive (HIV+) and HIV-negative (HIV-) patients. The cohort included nine treatment-naïve CRC patients (5 HIV+, 4 HIV-) who provided tumor-normal paired tissues collected from February 2021 to August 2022. All specimens were processed as independent biological replicates.

### Protein sample preparation

Frozen tumor-normal tissue pairs underwent mechanical fragmentation, phosphate-buffered saline perfusion for dehemoglobinization, and liquid nitrogen pulverization. Proteins were extracted using a standardized phenol-based method 13, quantified via bicinchoninic acid assay (Thermo Scientific), and assessed by SDS-PAGE. Lysates were aliquoted and stored at -80 °C for subsequent proteomic analysis.

### Trypsin enzymatic hydrolysis and TMT labeling

Protein aliquots (50 μg/sample) underwent sequential reduction-alkylation: reduction with 5 mM dithiothreitol (55 °C, 1 h) followed by alkylation using 10 mM iodoacetamide (room temperature, dark, 15 min). After acetone precipitation (8,000 ×g, 10 min, 4 °C), pellets were resuspended in 200 mM triethylammonium bicarbonate (TEAB) buffer. TPCK-treated trypsin digestion (1 mg/mL, 50:1 w/w) proceeded at 37 °C for 16 h. Resultant peptides were labeled with TMTpro reagent (Thermo Fisher) in 100 mM TEAB, and the reactions were quenched with 5% hydroxylamine (15 min).

### Reversed-phase liquid chromatography (RPLC) analysis

TMT-labeled peptides were fractionated on an EASY-nLC 1200 system (ThermoFisher) using an Agilent Zorbax Extend C18 column (2.1 × 150 mm, 5 µm) under alkaline conditions (pH 10). Separation was carried out through a binary gradient: mobile phase A: 2% acetonitrile/98% water, mobile phase B: 90% acetonitrile/10% water. A multi-step gradient (300 μL/min) was executed as follow: 0-8 min: 98% A (isocratic), 8-48 min: 95%→75% A, 48-60 min: 75%→60% A, 60-70 min: 10% A (re-equilibration). Eluates from 8-60 min were collected at 1-min interval into 15 fractions. The collected fractions were vacuum-concentrated prior to mass spectrometry (MS) analysis.

### Liquid chromatography-mass spectrometry (LC-MS/MS) analysis

Chromatographic separation employed a nano-UHPLC system with dual trap columns. Aliquots (5 μL) were loaded onto a PepMap100 trap column (100 μm×2 cm, C18; Thermo Scientific), then separated on a PepMap RSLC analytical column (75 μm×50 cm, C18) at 300 nL/min. The mobile phases consisted of (A) 0.1% formic acid in water and (B) 80% acetonitrile containing 0.1% formic acid. The following gradient was applied: 0-40 min: 2→28% B; 40-50 min: 28→42% B; 50-55 min: 42→90% B; and 55-75 min: 90% B (isocratic).

Mass spectrometric detection was carried out using a Q Exactive HF hybrid quadrupole-Orbitrap instrument (Thermo Fisher Scientific) in a positive mode. Full-scan MS spectra (m/z 350-1500) were acquired at 60k resolutions (automatic gain control (AGC) of 3e^6^, and maximum injection time (IT) of 50 ms). The top 20 most intense precursors were selected for HCD fragmentation (normalized collision energy (NCE) 32 eV). MS/MS spectra were recorded at 45k resolution (AGC 2e5, and max IT 80 ms) with 30 s dynamic exclusion.

### Protein identification

The LC-MS/MS raw data files (n = 30, representing 15 biological fractions, each injected in duplicate) were processed for proteomic analysis using Proteome Discoverer v2.4.1.15 (Thermo Fisher Scientific). Protein identification was performed via the Mascot algorithm (v2.3.02; Matrix Science) against the UniProt Human reference proteome database (release 2022.2.8, 9606 entries). Search parameters were set as follows: precursor mass tolerance of 10 ppm; fragment tolerance of 0.02 Da; trypsin digestion allowing up to two missed cleavages; fixed modifications for TMTpro 16-plex labeling (N-terminus/lysine) and carbamidomethylation (cysteine); and variable modifications for methionine oxidation and N-terminal acetylation. The instrument type was specified as Q Exactive HF. All datasets were deposited in the iProX public repository (accession: PXD043821) in accordance with proteomics community standards.

### Differential expression analysis

Differential expression analysis was restricted to the 6,840 proteins with valid TMT quantification data across all samples. The fold change (FC) in relative protein abundance between experimental groups was calculated, and statistical significance was assessed using an unpaired, two-sided Student's t-test.

Preliminary proteomic statistical evaluation was performed using Microsoft Excel 2010 (Microsoft Corporation). DEPs were defined by a fold change ≥ |1.5| and a p-value < 0.05. Advanced analyses—including volcano plots, Venn diagrams, and hierarchical clustering—were conducted using R (v4.2.2, R Foundation) 14. Four distinct DEP sets were identified through pairwise comparisons: DEPs-1: HIV+C vs HIV+A, DEPs-2: HIV-C vs HIV-A, DEPs-3: HIV+A vs HIV-A, and DEPs-4: HIV+C vs HIV-C. To specifically define HIV-associated cancer signatures, DEPs-1 was computationally refined by sequentially excluding proteins overlapping with DEPs-2, DEPs-3, and DEPs-4, resulting in the final DEPs-5 subset.

### Bioinformatics analysis of DEPs-5

The DEPs-5 subset was functionally annotated using the STRING database (https://string-db.org/), which incorporates Kyoto Encyclopedia of Genes and Genomes (KEGG) pathway data (http://www.genome.jp/kegg/genes.html). Molecular interaction networks and pathway maps were generated by extracting KEGG annotations and visualizing the network in Cytoscape (v3.10.0-BETA1) using STRING-derived node attributes. Further processing within Cytoscape included: PPI submodule screening with MCODE plugin 15 (criteria: degree cutoff = 2, node score = 0.2, k-core = 2, max depth = 100, min genes = 4), and hub gene identification using cytoHubba plugin 16 via MCC-based node ranking.

To validate clinical relevance, 115 pathway-enriched DEPs-5 were cross-referenced with colorectal cancer (COAD) proteogenomic data in the CPTAC database (cProSite; https://cprosite.ccr.cancer.gov/) 17. Additionally, survival analysis was performed on HIV-associated proteins overlapping with these five pathways using the Cancer Genome Atlas (TCGA) database via the GEPIA2 portal (http://gepia2.cancer-pku.cn/#survival). This analysis evaluated correlations between protein expression and survival in COAD cohorts.

### Immunohistochemical staining

Given the association between HIV infection and poorer CRC prognosis, coupled with reduced survival in CRC patients exhibiting low *SERPINA1* expression, we conducted immunohistochemical validation of *SERPINA1*—a downregulated protein identified in complement and coagulation cascades pathway (CCC pathway). Nine CRC/adjacent tissue pairs were stained with *SERPINA1* antibody (Anti-alpha 1 Antitrypsin [EPR9090], ab166610) on a BOND RX Research Stainer (Leica Biosystems). Stained sections were scanned at 100× or 400× magnification (Olympus BX40 microscope with logenEPAS9000). For semi-quantitative analysis, ten random 400× fields per slide were imaged. *SERPINA1*-positive cells were counted per field, with expression change calculated as the ratio of average positive cells in cancer tissue versus adjacent tissue, stratified by HIV infection status.

### Western blotting

To validate proteomic results and the involvement of the CCC pathway, we analyzed *C8B*, a downregulated gene that serves as a terminal component in the endpoint of the CCC pathway, by western blot. Protein extracts (20 µg) from nine paired tumor/adjacent normal colon tissues underwent SDS-PAGE separation and PVDF membrane transfer (0.45 µm, Millipore). Membranes were blocked with 10% skim milk for 60 min at room temperature or overnight at 4 °C), followed by incubation with a rabbit anti-C8B primary antibody (EPR23764-1, 1:1000, Abcam) overnight at 4 °C. After washing with TBST, the membranes were incubated with an HRP-conjugated goat anti-rabbit IgG secondary antibody (074-1506, 1:5000, KPL) for 1 h at room temperature. Signals were detected by ECL, imaged using a ChemiScope 5300 (Clinx), and quantified via ImageJ software (v1.51j8, NIH). The data represent three independent biological replicates, each measured in two technical replicates during Image J quantification.

### Statistical analysis

In the proteomic analysis, DEPs were identified using Excel 2016 with dual thresholds: a |fold change| ≥ 1.5 and a* p* < 0.05 from an unpaired Student's t-test.

For western blot and immunohistochemical (IHC) data, statistical analyses were performed using GraphPad Prism 9.0. Unpaired t-tests were used for comparisons between two groups, and one-way ANOVA was used for comparisons among multiple groups. A *p* < 0.05 was considered statistically significant.

## Results

### The characteristics of patients

Table 1 summarizes the baseline characteristics of nine enrolled male patients (5 HIV+, 4 HIV-). The mean age was 57.75 years for HIV- and 52.4 years for HIV+. All tumors were histologically confirmed as ulcerative adenocarcinomas. Lymph node metastasis was identified in four cases, with two cases in each group.

### Overview of proteomic study in this study

**Figure 1:**
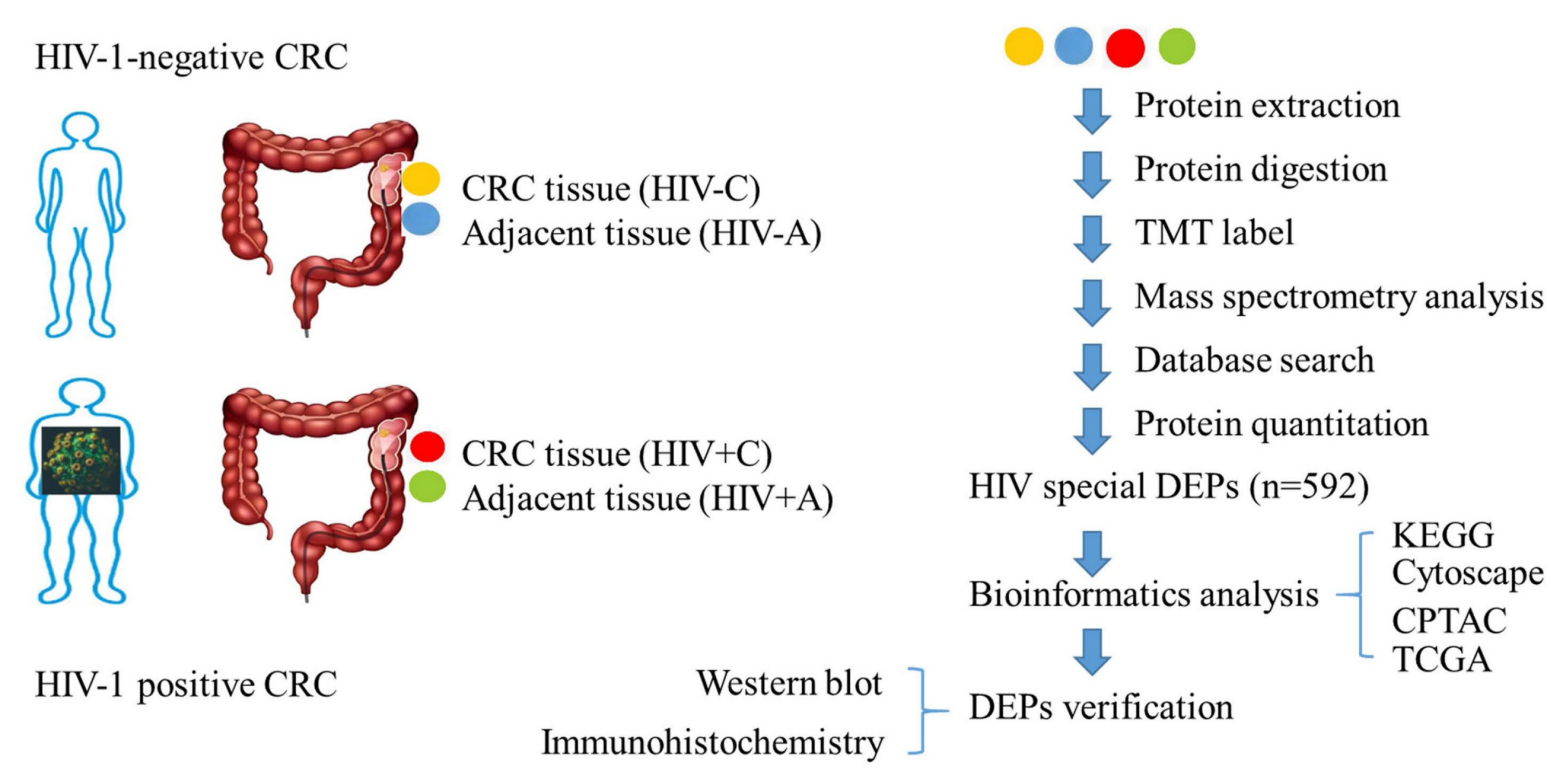
The overview of proteomic study.

To decipher HIV's influence on colon tissue pathophysiology and its CRC-associated mechanisms, we conducted TMT proteomic profiling of tumor/adjacent tissues from HIV+ and HIV- CRC cohorts. Bioinformatics analysis of HIV-related DEPs (DEPs-5 subset) revealed candidate proteins *C8B* and *SERPINA1*, which underwent western blot and IHC verification (Figure 1).

### Basic information for protein identification

**Figure 2:**
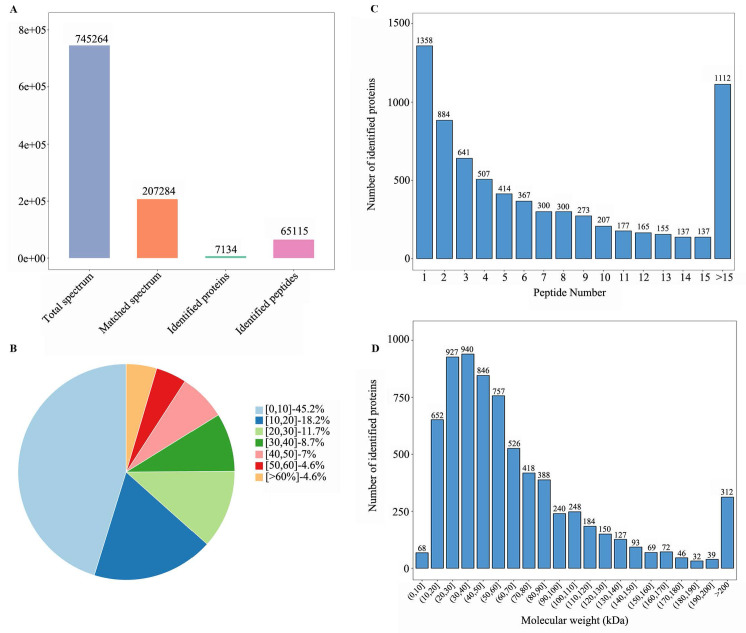
Qualitative analysis of identified proteins. **(A)** Spectra, peptides, and protein quantification summary. **(B)** Protein distribution by peptide count. **(C)** Sequence coverage distribution. **(D)** Molecular weight distribution (kDa).

TMT-based proteomic profiling identified 745,262 mass spectra, with 207,284 matched spectra mapping to 65,115 unique peptides and 7,134 proteins (Figure 2A). Precise quantification of 6,840 proteins was achieved via TMT tags (Table S1). Subsequent characterization of the proteome showed that the sequence coverage was 0-10% for 45% of the proteins and 10-20% for 18.2% of them (Figure 2B). The number of peptides identified per protein ranged from 1 to over 15 (Figure 2C), and the molecular weight of the majority of proteins fell between 0 and 200 kDa (Figure 2D).

### Identification of DEPs

Four sample groups were analyzed: colon tumors and adjacent normal tissues from individuals with or without HIV-1 infection. Comparative proteomics revealed 749 DEPs in HIV-positive tissues (DEPs-1: 544 upregulated and 205 downregulated; Table S2) and 431 DEPs in HIV-negative tissues (DEPs-2: 386 upregulated and 45 downregulated; Table S3). The common DEPs between DEPs-1 and DEPs-2 were 154, including 134 upregulated and 20 downregulated proteins (Table S4).

**Figure 3:**
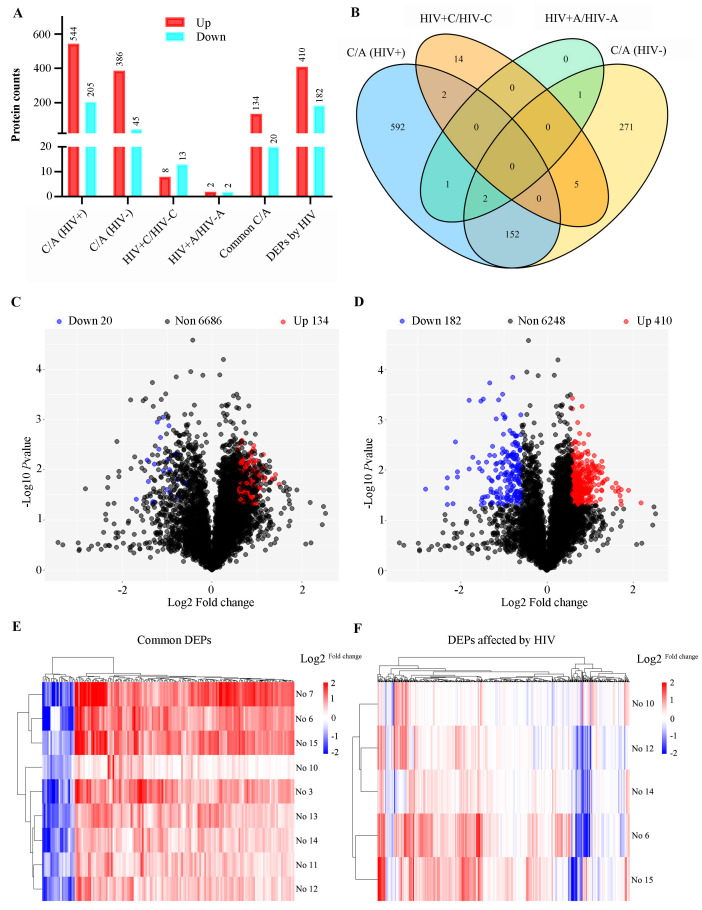
**Bioinformatics analysis of differentially expressed proteins (DEPs)**. **(A)** Number of DEPs in different comparisons. **(B)** the Venn plot of different comparisons; HIV+, HIV-, represent HIV infection, and no HIV infection, respectively. C/A: cancerous/adjacent tissues. **(C)** Volcano plot of common DEPs. **(D)** Volcano plot of HIV-specific DEPs (DEPs-5). **(E)** Heatmap of common DEPs. **(F)** Heatmap of HIV-specific proteins (DEPs-5). Sample annotation: HIV-: #3,7,11,13; HIV+: #6,10,12,14,15. Log 2^fold change^ was shown in color scale (red > 0, blue < 0).

To discover HIV-specific CRC signatures, the DEPs-1 set was sequentially filtered to remove proteins overlapping with those in DEPs-2, DEPs-3 (n = 21; Table S5), and DEPs-4 (n = 4; Table S6), resulting in 592 HIV-associated DEPs (410 upregulated, 182 downregulated; Table S7). Across all comparisons, upregulated proteins significantly outnumbered downregulated ones (Table 2, Figure 3A).

The characteristics and commonality of DEPs from the four comparisons were visualized using a Venn diagram (Figure 3B). The distribution of DEPs with a fold change between 1.5 and 3.0 was displayed in volcano plots for both the shared (Figure 3C) and the HIV-specific (Figure 3D) protein sets. Furthermore, a clustering heatmap illustrated the expression patters of the DEPs, highlighting the substantial divergence between tumor and adjacent normal tissues for both the common proteins (Figure 3E) and the HIV-specific proteins (Figure 3F).

### Bioinformatic analysis of DEPs-5

**Figure 4:**
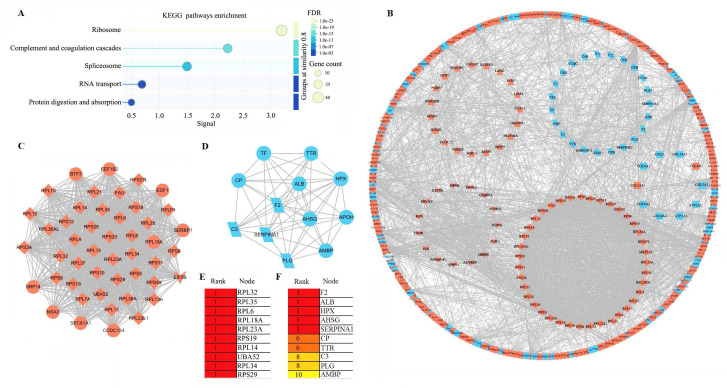
** Protein-protein interaction (PPI) network analysis**. **(A)** KEGG pathways enrichment of 592 HIV-CRC-specific DEPs (DEPS-5). **(B)** PPI network of the 592 proteins in DEPs-5. Proteins are shape-coded by their primary enriched pathway: diamond (ribosome), parallelogram (complement and coagulation cascades), triangle (spliceosome), inverted triangle (RNA transport), and rectangle (protein digestion and absorption). Red and blue backgrounds denote upregulated and downregulated proteins, respectively. **(C)** MCODE1 module identified by MCODE analysis in Cytoscape. **(D)** MCODE2 module identified by MCODE analysis in Cytoscape. **(E)** Top 10 hub proteins in MCODE1 identified by cytoHubba. **(F)** Top 10 hub proteins in MCODE2 identified by cytoHubba.

In this study, we specifically focused on the HIV-associated DEPs, designated as DEPs-5, and identified a total of 592 HIV-CRC-specific DEPs. Functional analyses revealed significant enrichment of these DEPs in five pathways (Table S8 and Figure 4A). Ribosome-associated DEPs (n = 40, all upregulated) constituted the largest group, followed by those in CCC pathway (n = 24, all downregulated), spliceosome (n = 24, all upregulated), RNA transport (n = 18, all upregulated), and protein digestion and absorption (n = 12; 10 downregulated and 2 upregulated). In total, 115 non-redundant genes were involved in these five pathways.

Protein-protein interaction analysis further clustered 39 ribosomal proteins, 23 from CCC pathway, 19 spliceosomal proteins, and 15 RNA transport proteins into distinct modules (Figure 4B). To identify hub genes, we performed MCODE and cytoHubba analyses within Cytoscape. MCODE1 was comprised primarily of ribosomal proteins (Figure 4C), while MCODE2 was dominated by proteins from CCC pathway (Figure 4D). The top 10 hub genes in MCODE1 included *RPL32* and *RPL6*, among others (Figure 4E), whereas the top 10 in MCODE2 included *F2* and *C3* (Figure 4F).

### Analysis of the DEPs through public databases

**Figure 5:**
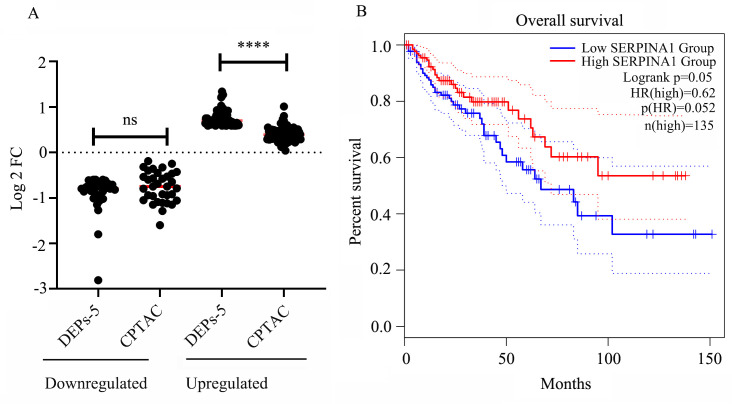
Validation of DEPs-5 expression and survival analysis. (A) Comparison of gene expression levels for DEPs-5 between our dataset and the CPTAC database (Tumor Count (n = 97), Adj. Normal Count (n = 100)). (B) Association between *SERPINA1* expression levels and patient survival.

We examined the expression of the 115 HIV-specific DEPs (excluding 3 replicates) within KEGG pathways using the CPTAC database. A comparing of the DEPs-5 expression profiles with CPTAC data revealed generally consistent expression trends. However, proteins upregulated in DEPs-5 showed relatively small fold changes (FC) in the CPTAC dataset (mean FC = 1.34, p < 0.05), all below 1.5. Collectively, these results indicate that HIV infection significantly increases the expression of upregulated genes, with FC of 1.70 in HIV+C vs HIV+A, and 1.34 from CPTAC, respectively. Similarly, the downregulated proteins also exhibited decreased expression, with an average FC of 0.61 in the CPTAC dataset and 0.55 in our HIV+ cancer versus adjacent tissues comparison (*p* = 0.13) (Table S8 and Figure 5A).

### Analysis of the relationship between patient survival and gene expression

Survival analysis using TCGA data revealed that four genes of the 115 KEGG-enriched genes were significantly associated with survival in patients with colon adenocarcinoma (COAD). These four genes were *SERPINA1* and *C3* (both downregulated), and *ALYREF* and *COL8A1* (both upregulated) (Figure S1). Higher expression of *C3* and *COL8A1* correlated with a worse prognosis, while lower expression of *SERPINA1* (Figure 5B) and *ALYREF* was associated with poorer outcomes.

### Immunohistochemical validation of *SERPINA1* expression

**Figure 6:**
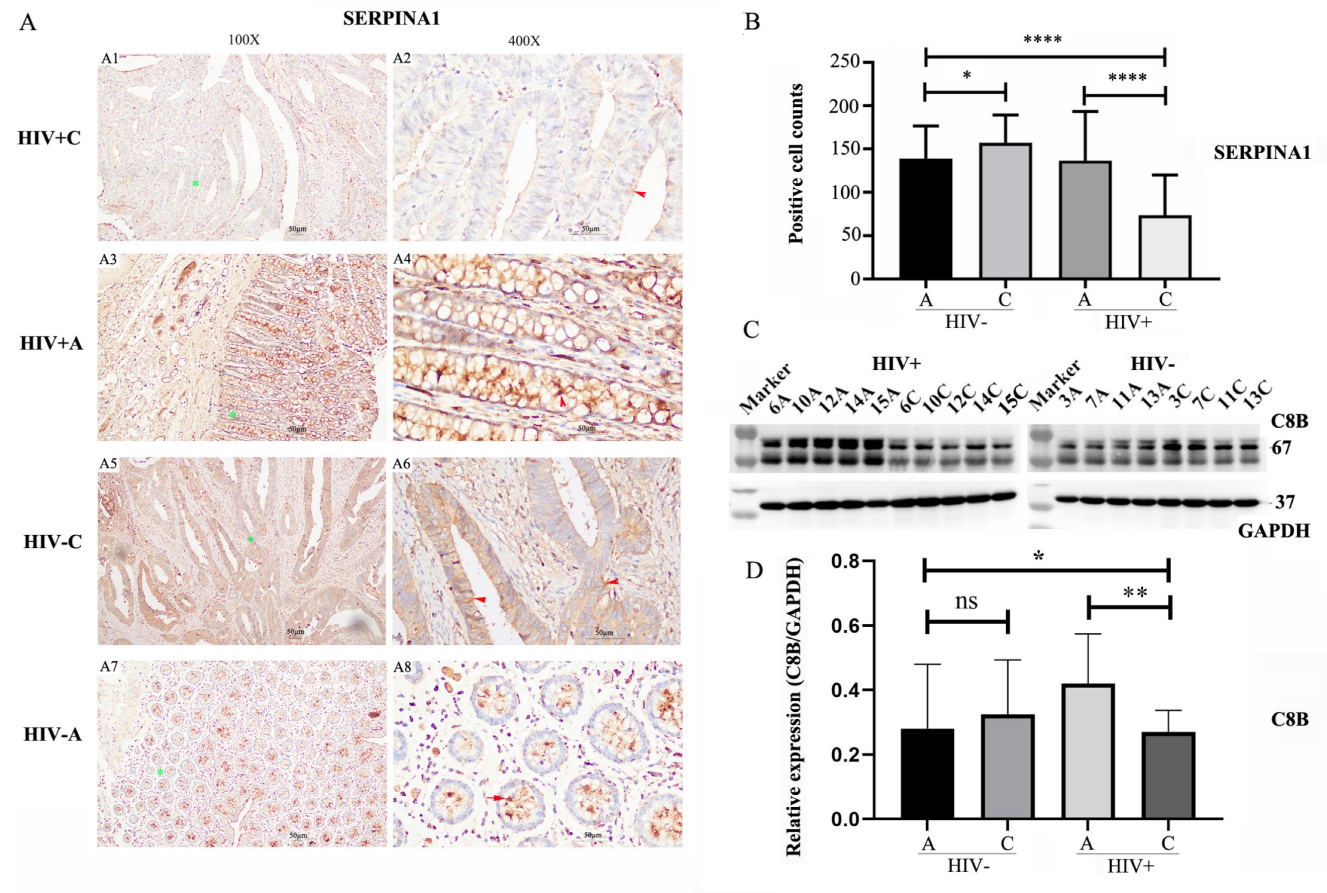
** Immunohistochemical and Western Blot Analysis of *SERPINA1* and *C8B* Expression**. **(A)** Immunohistochemical (IHC) staining of *SERPINA1* in colorectal tissues. Representative images are shown at 100X and 400X magnifications. Panels A1-A2: Tumor tissue from HIV-positive patients (HIV+C). Panels A3-A4: Adjacent non-tumor tissue from HIV-positive patients (HIV+A). Panels A5-A6: Tumor tissue from HIV-negative patients (HIV-C). Panels A7-A8: Adjacent non-tumor tissue from HIV-negative patients (HIV-A). Green stars denote areas magnified from 100X to 400X. Brown staining indicates positive *SERPINA1*; nuclei are counterstained with DAPI (blue). Red arrows highlight *SERPINA1*-positive cells. **(B)** Semi-quantitative analysis of *SERPINA1* IHC staining. Positive cell counts per field are presented for HIV-C (n=4 patients, 10 fields/slice), HIV-A (n=4, 10 fields/patient), HIV+C (n=5, 10 fields/patient), and HIV+A (n=5, 10 fields/patient). Data represent mean ± SD. Statistical significance was determined by unpaired Student's t-test. **(C)** Western Blot (WB) analysis of *C8B* expression in paired colon tumor (C) and adjacent non-tumor (A) tissues. Left panel: Samples from HIV-positive patients (C: 6C, 10C, 12C, 14C, 15C; A: 6A, 10A, 12A, 14A, 15A). Right panel: Samples from HIV-negative patients (C: 3C, 7C, 11C, 13C; A: 3A, 7A, 11A, 13A). **(D)** Quantification of *C8B* protein levels by WB. Band intensities were measured using ImageJ software. Data are presented as mean ± SD. Statistical significance was assessed by unpaired Student's t-test. ns: not significant, p > 0.05; *p < 0.05; **p < 0.01; ***p < 0.005; ****p < 0.001.

Given the association between HIV infection and poorer prognosis in colorectal cancer (CRC), coupled with the reports of reduced survival in CRC patients exhibiting lower *SERPINA1* expression, we conducted IHC analysis to verify *SERPINA1* protein levels (Figure 6A). IHC staining revealed a significant downregulation of *SERPINA1* in tumor tissues from HIV-positive patients (Figure 6A1, A2) compared to their adjacent non-tumor tissues (Figure 6A3, A4). Conversely, in HIV-negative patients, cancer tissues displayed elevated *SERPINA1* expression (Figure 6A5, A6) relative to matched adjacent controls (Figure 6A7, A8). Semi-quantitative assessment of IHC-positive cells (Figure 6B) yielded average counts (±SD) of 73.4 ± 46.8 for HIV+C, 136.8 ± 56.7 for HIV+A, 157.5 ± 31.7 for HIV-C, and 139.4 ± 37.5 for HIV-A. This analysis confirmed an approximately two-fold reduction in *SERPINA1*-positive cells in HIV+C versus HIV+A (p < 0.0001). In stark contrast, HIV-C exhibited only a modest, albeit significant, increase (~1.13-fold) compared to HIV-A (p < 0.05).

### Verification of *C8B* expression by western blot (WB)

To verify the proteomics results and the finding from the bioinformatics analysis, we performed WB to validate *C8B* (a downregulated protein). Results demonstrated significantly decreased *C8B* levels in HIV+C vs. HIV+A tissues (Fig. 6C-D), while, no significant difference was observed in HIV-negative tissues (HIV-C vs. HIV-A).

## Discussion

Individuals with HIV face elevated cancer risks, worse clinical progression, and reduced survival compared to HIV-negative populations 18-20. Malignancies account for 10-20% of all deaths in individuals with HIV 18-22. Although CRC belongs to non-AIDS defining cancers 23, HIV-positive individuals constitute 7.7% of the CRC patient population and experience an earlier onset of the disease 24. These observations underscore the need to elucidate HIV-specific oncogenic mechanisms in CRC.

Our TMT-based proteomic analysis of HIV-positive (HIV+CRC) and HIV-negative (HIV-CRC) tumors represents a significant methodological advance. So far, only one study has examined HIV+CRC proteomes using data-independent acquisition MS, identifying 467 DEPs in tumors versus adjacent tissue (HIV+C vs HIV+A), in which, the proteomic data for non-HIV-CRC (HIV-CRC) were sourced from CPTAC dataset 25. In our study, HIV+C, HIV-C and their adjacent controls were included, and 6,840 proteins were quantified. 749 DEPs specific to HIV+CRC (DEPs-5) were detected. Cross-referencing revealed 72 overlapping DEPs with 98.6% expression concordance (Table S8), confirming the high reproducibility of both studies. Furthermore, our dataset was about twice as large as that in the previous study 25, in both the total proteins identified and the number of DEPs.

Focusing on HIV-specific DEPs (DEPs-5, n = 592), we found that ribosome-associated proteins (n = 40) represented the most significantly upregulated pathway. Ribosomal biogenesis supports viral replication through HIV-mediated host machinery hijacking 26, 27 and fuels carcinogenesis via mTOR/Myc-driven neogenesis 28. In contrast, proteins in the CCC pathway showed marked downregulation (24 proteins). As the complement system comprises critical immune regulators, its deficiency impairs pathogen clearance and antibody responses, potentially facilitating both HIV persistence and CRC progression 28-33.

Since DEPs-5 are related to HIV-associated CRC, we furthermore compared the gene expression of the DEPs (enriched in the five pathways) with that from the CPTAC database. Results revealed HIV infection consistently amplified activities in ribosome, spliceosome, and RNA transport pathways while attenuating the complement/coagulation cascade and protein digestion pathways. This dual mechanism—viral exploitation of translational machinery coupled with complement paralysis—likely drives accelerated CRC pathogenesis in HIV infection.

Finally, we verified the downregulation of *C8B* and* SERPINA1*. *C8B* was reduced in HIV+CRC tumors, consistent with observations in hepatocellular carcinoma (HCC) where low *C8B* expression correlates with malignant progression and poor prognosis 34, 35.* SERPINA1* was also downregulated in HIV-associated CRC, contrasting with reports of its elevated expression and association with poor outcomes in non-HIV CRC 36. As a protease inhibitor, *SERPINA1* plays a vital role in tissue protection by neutralizing serine proteinases; its deficiency is known to contribute to emphysema and degenerative diseases 37. According to the HIV-1-human interaction database, *SERPINA1* (which encodes alpha-1-antitrypsin, AAT) can interact with HIV proteins such as gp160 and Pr55(Gag), and may compete with gp120-proteinase binding 38. AAT has been implicated in HIV infection 39. Both AAT and its C-terminal peptide, VIRIP, exhibit anti-HIV activity 40. Moreover, AAT therapy has demonstrated anti-inflammatory and immunomodulatory properties 41. Reduced *SERPINA1* expression may compromise AAT-mediated tissue protection against proteolytic enzymes, disrupting the balance between inflammation, physiological repair, and chronic injury 42. *SERPINA1* deficiency has also been associated with HIV disease progression 39 and CD4⁺ T-cell loss in PLWH 43. Taken together, in HIV-CRC, reduced SERPINA1 may promote colorectal carcinogenesis and worsen prognosis through both enhanced HIV-induced inflammation and diminished host immunomodulation. In summary, the coordinated downregulation of *C8B* and *SERPINA1* in HIV⁺CRC points to a potential virally driven suppression mechanism. This dual reduction may act synergistically to impair HIV clearance and complement-dependent immunity, ultimately leading to poorer clinical outcomes 33.

This study has several limitations. First, the sample size was small (n = 9), and future work would benefit from a larger cohort or multicenter validation to strengthen the findings. Second, the validation was limited to only two proteins. Furthermore, the functional roles of key DEPs like *C8B* and *SERPINA1* remain unexplored, as no knockout or gene-silencing experiments were conducted. Finally, the correlation between the expression levels of these proteins and the patients' pathoclinical features was not experimentally verified.

In summary, our TMT-based quantitative proteomics revealed 592 distinctly altered proteins in HIV-infected colorectal cancer (HIV+CRC) compared to adjacent tissues. These DEPs demonstrated significant enrichment in five key pathways: ribosome biogenesis, complement/coagulation cascades, spliceosome assembly, RNA transport, and protein digestion/absorption. We propose a dual pathogenic mechanism wherein HIV hijacks ribosomal machinery to facilitate viral replication while suppressing complement activity to promote tumorigenesis. The downregulated effectors *C8B* and *SERPINA1* emerge as promising therapeutic targets for mitigating HIV-driven CRC progression.

## Supplementary Material

Supplementary figures and tables.

## Figures and Tables

**Table 1:** Baseline characteristics and TMT labeling information of the study patients.

No.	Infection	Age	Sex	Transfer	Samples	Label
3	HIV-	68	male	lymph node metastasis	Adjacent (HIV-A)	126
					Cancer (HIV-C)	128C
7	HIV-	47	male	lymph node metastasis	Adjacent (HIV-A)	127N
					Cancer (HIV-C)	129N
11	HIV-	68	Male	Not	Adjacent (HIV-A)	127C
					Cancer (HIV-C)	129C
13	HIV-	48	Male	Not	Adjacent (HIV-A)	128N
					Cancer (HIV-C)	130N
6	HIV+	36	Male	lymph node metastasis	Adjacent (HIV+A)	130C
					Cancer (HIV+C)	133N
10	HIV+	39	Male	lymph node metastasis	Adjacent (HIV+A)	131N
					Cancer (HIV+C)	133C
12	HIV+	63	Male	Not	Adjacent (HIV+A)	131C
					Cancer (HIV+C)	134N
14	HIV+	62	Male	Not	Adjacent (HIV+A)	132N
					Cancer (HIV+C)	134C
15	HIV+	62	Male	Not	Adjacent (HIV+A)	132C
					Cancer (HIV+C)	135N

*Note*: The patient identification number (No.) corresponds to the storage number in our specimen bank.

**Table 2:** The number of DEPs in different comparisons.

Comparison	Total DEPs	Up	Down	Name
HIV+C vs HIV+A	749	544	205	DEPs-1
HIV-C vs HIV-A	431	386	45	DEPs-2
HIV+A vs HIV-A	4	2	2	DEPs-3
HIV+C vs HIV-C	21	8	13	DEPs-4
Common C vs A with or without HIV	154	134	20	Common DEPs
DEPs affected by HIV (DEPs 1-2-3-4)	592	410	182	DEPs-5
