# Supplementary figures and images for: Complement and Coagulation Cascades Pathway was Inactivated in HIV-Associated Colorectal Cancer: Results from a Proteomics Study
Source: J Cancer. 2026 Jan 14;17(2):427–38. doi: 10.7150/jca.124804 (PMC12825422; doi:10.7150/jca.124804)

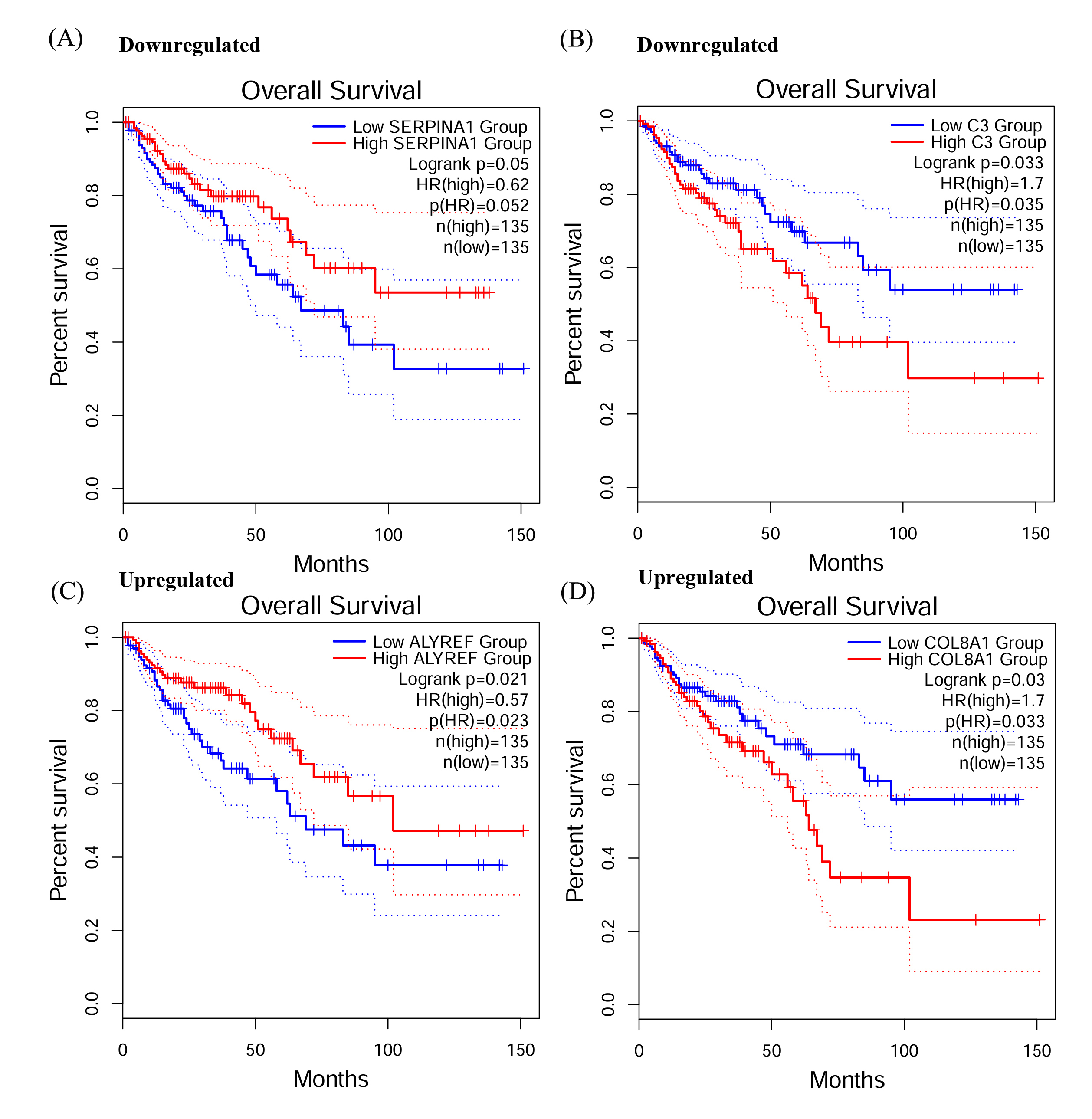

Supplement: Supplementary file 1 — Supplementary figures and tables. [file jcav17p0427s1.zip › Supplement figure and tables-20250813/Figure S1-20250807.jpg]
